# Clinical and metabolic profile of adults with obesity attending lifestyle medicine clinics

**DOI:** 10.1371/journal.pone.0342153

**Published:** 2026-02-02

**Authors:** Hanan N. AlTaib, Reem AlAqeel, Amr A. Arafat, Faisal Kunnathodi, Abdulmajeed AlShaikh, Haifa F. AlOtaibi

**Affiliations:** 1 Department of Family Medicine, Prince Sultan Military Medical City, Riyadh, Saudi Arabia; 2 Health Research Center, Ministry of Defense Health Services, Riyadh, Saudi Arabia; 3 Department of Adult Cardiac Surgery, Prince Sultan Cardiac Center, Riyadh, Saudi Arabia; University of Diyala College of Medicine, IRAQ

## Abstract

**Background:**

Lifestyle Medicine Clinics (LMCs) in primary care provide structured, multidisciplinary obesity care, but evidence from Saudi Arabia on patient profiles and the effectiveness of pharmacologic therapy in this setting is limited.

**Methods:**

We conducted a retrospective cohort study at a primary health care center in Prince Sultan Military Medical City, Riyadh (2023–2024). Adults aged 18–75 years with Body Mass Index (BMI) 30–40 kg/m² and at least one obesity-related comorbidity were included. Patients received either Liraglutide plus lifestyle modification or lifestyle modification alone. Data from the LMCs electronic registry were analyzed after 1:1 propensity score matching. Within-group changes were evaluated using paired tests; between-group differences over time were analyzed using repeated-measures ANOVA.

**Results:**

Among 664 patients (299 receiving Liraglutide and 365 receiving lifestyle modification alone), 280 matched pairs were analyzed (median age 40 years; 73% female). Both groups achieved significant within-group reductions in BMI, systolic and diastolic blood pressure, and HbA1c (all p < 0.05). Between-group comparisons showed a statistically significant difference only in waist circumference change (p = 0.03). Mean BMI change was − 0.75 kg/m² with Liraglutide versus − 0.71 kg/m² without Liraglutide (p = 0.939). No significant between-group differences were observed for glycemic control, blood pressure, or lipid parameters.

**Conclusions:**

In a real-world primary care setting, Liraglutide plus lifestyle modification and lifestyle modification alone both produced a substantial benefit in metabolic and anthropometric parameters. The incremental benefit of Liraglutide was confined to a modest reduction in waist circumference and did not translate into clinically meaningful differences compared with lifestyle intervention alone. Structured, multidisciplinary lifestyle care should remain the foundation of obesity management, with pharmacotherapy targeted to selected patients.

## Introduction

Global obesity prevalence continues to rise despite public health initiatives aimed at prevention [1]. In Saudi Arabia, recent estimates indicate an overall prevalence of 33.7%, with higher rates among women (39.5%) than men (29.5%) [2].

Obesity has a detrimental impact on health, economic productivity, and overall quality of life. It is associated with increased risk of metabolic, mechanical, neoplastic, and mental health disorders [3,4]. The rising prevalence imposes substantial direct and indirect economic costs [4].

Lifestyle modification, including dietary change, increased physical activity, adequate sleep, stress management, avoidance of harmful substances, and social support, is the cornerstone of obesity management [5]. Pharmacologic therapy is an important adjunct for patients with substantial weight-related health risks or inadequate response to lifestyle measures alone [6].

In Saudi Arabia, studies have examined dietary habits [7–10], physical inactivity [11,12], the effectiveness of intensive lifestyle interventions [12,13], and pharmacological treatments [14–17]. Most of this research has been conducted in non–primary care settings [12–14].

Primary health care centers, however, are the first point of contact for most patients. Understanding the clinical profile of adults with obesity and strengthening obesity management in primary care are key strategies for addressing the epidemic [17].

Lifestyle Medicine Clinics (LMCs) within primary care are providing comprehensive, evidence-based interventions focused on lifestyle modifications. However, there is a scarcity of research examining the specific characteristics of adults with obesity attending LMCs in Saudi Arabia and the services these clinics offer.

Although obesity is a global epidemic, health system responses vary widely [1]. Most evidence on pharmacologic therapy for obesity derives from randomized clinical trials in controlled environments, predominantly in Western populations [18]. These findings may not reflect outcomes in routine practice, particularly in health systems such as Saudi Arabia’s, where primary care serves as the first point of contact [19] and cultural factors, gender differences, and comorbidity patterns differ substantially [20]. Studying obesity care in this setting, therefore, offers insights that are both locally relevant and internationally generalizable [21].

To address two complementary objectives, this study first describes the demographic, anthropometric, clinical, and behavioral profile of adults with obesity attending LMCs. A secondary, comparative analysis evaluates changes in selected clinical and metabolic outcomes between patients who received Liraglutide in addition to lifestyle intervention and those who received lifestyle intervention alone. This study will provide valuable evidence on the needs of adults with obesity seeking care in LMCs and inform the development of targeted interventions to improve obesity management within the primary care setting.

### Objectives

To describe the demographic (age, sex), anthropometric (body weight, height, Body Mass Index [BMI], waist circumference), clinical (presence of chronic diseases), and behavioral (dietary habits, physical activity, smoking status, sleep duration, mental health screening) characteristics of adults with obesity attending LMCs in primary health care.

To compare changes in key clinical and metabolic outcomes between patients receiving Liraglutide plus lifestyle modification and those receiving lifestyle modification alone. Outcomes include BMI, body weight, blood pressure, glycemic control (HbA1c, fasting blood glucose), waist circumference, lipid profile (total cholesterol, LDL-C, HDL-C, triglycerides), and other relevant metabolic markers.

## Methods

### Study design and setting

We conducted a retrospective cohort study of patients referred to LMCs within the primary care system, reported in accordance with the STROBE Statement for observational studies [22]. Consecutive patients referred to LMCs at Prince Sultan Military Medical City (PSMMC), Riyadh, Saudi Arabia, between January 2023 and December 2024, were eligible for inclusion. The PSMMC primary care system provides services to military personnel and their dependents and delivers comprehensive obesity care through LMCs. The LMC is a structured, multidisciplinary primary care service designed to deliver comprehensive obesity management. Core components include individualized dietary counseling by trained dietitians, physical activity prescription, behavioral and motivational counseling, sleep and stress management guidance, smoking cessation support, and regular monitoring of anthropometric and metabolic parameters. Care is delivered by a multidisciplinary team consisting of family physicians, dietitians, nurses, and health educators. Patients are typically scheduled for follow-up visits every 3–6 months. In the matched cohort, the median duration between baseline and follow-up assessments was 3 months (interquartile range: 2–9 months). Standard lifestyle recommendations included written material and a 20- to 30-minute individualized counseling session with a dietitian covering diet, physical activity, and behavior change strategies. Patients were advised to follow the Saudi Food Guide Pyramid [23] and to engage in at least 150 minutes of moderate-intensity physical activity per week.

#### Participants.

Eligible participants were adults aged 18–75 years with a BMI between 30 and 40 kg/m² and a diagnosis of at least one of the following: prediabetes, hypertension, hyperlipidemia, hypothyroidism, metabolic dysfunction–associated steatohepatitis (MASH), or bronchial asthma. This study primarily included adults with primary (simple) obesity attending LMCs. Hypothyroidism was included only when patients were on stable thyroid hormone replacement therapy with documented euthyroid status, reflecting routine primary care practice.

LMCs manage patients with stable chronic disease and obesity up to a BMI of 40 kg/m²; individuals with higher BMI or unstable chronic conditions are referred for advanced therapies. This policy reflects safety considerations and ensures that patients requiring pharmacologic, surgical, or specialized multidisciplinary management beyond the scope of LMCs receive appropriate care. We excluded patients with BMI > 40 kg/m², type 1 or type 2 diabetes mellitus, chronic liver disease, chronic kidney disease, heart failure, obstructive sleep apnea, or severe psychiatric illness (defined as a documented ICD-10 diagnosis requiring ongoing psychiatric treatment). Patients who were on anti-obesity pharmacotherapy at baseline were also excluded.

#### Participant Selection.

**Fig 1:**
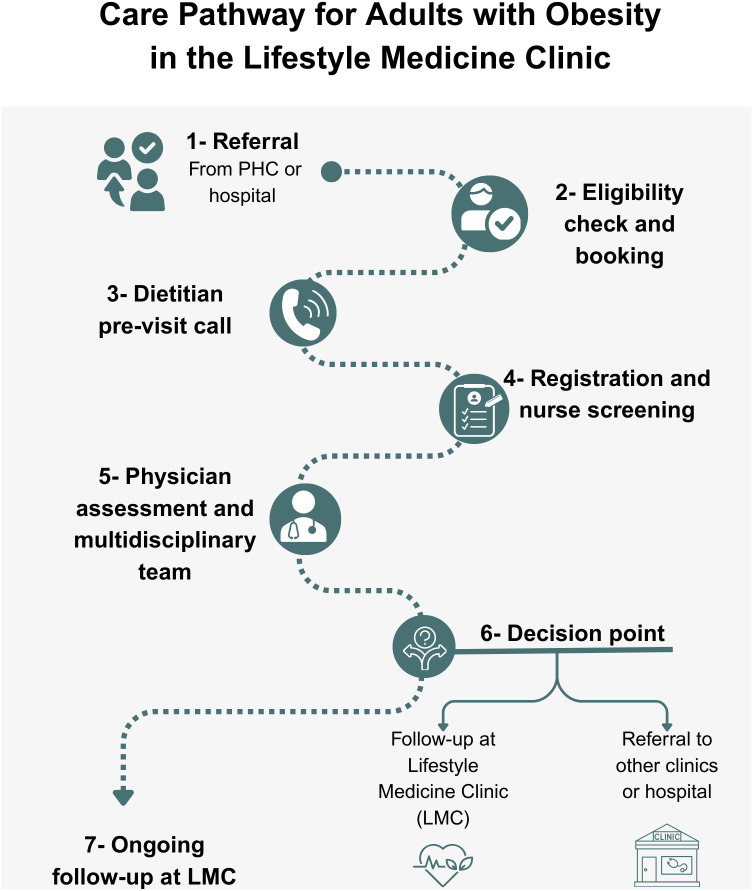
Care pathway for adults with obesity in the Lifestyle Medicine clinic. Referral from primary health care or hospital is followed by eligibility screening, registration, nurse assessment, dietitian pre-visit counseling, and physician evaluation. At the decision point, patients continue follow-up at the Lifestyle Medicine Clinic or are referred to other clinics or hospitals as appropriate.

Patients were referred to LMCs from primary health care clinics (Fig 1). After eligibility screening, participants underwent 3–4 multidisciplinary sessions over the follow-up period, addressing diet, exercise, and behavior modification. Although lifestyle modification is the foundation of care in LMCs, pharmacologic therapy is considered an adjunct for selected patients who meet clinical eligibility criteria and have obesity-related comorbidities or an inadequate response to lifestyle intervention alone. In this context, Liraglutide is prescribed as part of an integrated obesity management strategy, in accordance with international clinical guidelines, and is always combined with ongoing lifestyle counseling rather than used as monotherapy [24]. Anti-obesity pharmacotherapy, including Liraglutide, was prescribed during follow-up on the basis of clinical judgment. When pharmacologic therapy was initiated, Liraglutide was prescribed according to routine clinical practice for obesity management. Treatment was initiated at a dose of 0.6 mg once daily, with weekly dose escalation in 0.6 mg increments as tolerated, to a maximum maintenance dose of 3.0 mg daily. Dose titration was individualized based on gastrointestinal tolerability and clinical response.

### Data collection

Data were extracted from the PSMMC LMC electronic registry, which captures demographics, medical history, laboratory results, and anthropometric measurements. The data extraction was carried out between April 28, 2025, and June 28, 2025. Variable definitions are detailed in the S1 Table. Validated instruments were used when available: physical activity was recorded according to the International Physical Activity Questionnaire (IPAQ) [25], depressive symptoms using the Patient Health Questionnaire-9 (PHQ-9) [26], and smoking status from structured intake forms. Laboratory tests, including HbA1c, lipid profile, alanine aminotransferase (ALT), and vitamin D, were performed in the PSMMC central laboratory using standardized assays. Data were entered into a standardized case-report form and imported into Stata software (StataCorp. Stata Statistical Software: Release 18. College Station, TX: StataCorp LLC; 2023) [27]. To maintain confidentiality, the authors had no access to any information that could identify individual participants during or after data collection. Quality control included completeness checks and review of outliers.

Baseline variables included age, sex, weight, height, BMI, waist circumference, comorbidities, Edmonton Obesity Staging System (EOSS) score, and smoking status. Follow-up variables included BMI, waist circumference, blood pressure, HbA1c, LDL, HDL, triglycerides, ALT, vitamin D, physical activity, dietary pattern, and depressive symptoms.

#### Statistical analysis.

Analyses were conducted with Stata software [27]. Descriptive statistics are reported as mean ± standard deviation (SD) or median (interquartile range [IQR]) for continuous variables and as number (percentage) for categorical variables.

To reduce confounding, we performed 1:1 propensity-score matching using nearest-neighbor methods without replacement. The propensity model included age, sex, baseline BMI, waist circumference, EOSS score, smoking status, and comorbidities. Covariate balance was assessed with standardized mean differences (SMD), with values <0.10 indicating adequate balance.

Between-group comparisons at baseline were performed using independent-samples t-tests or Mann–Whitney U tests for continuous variables and chi-square or Fisher’s exact tests for categorical variables. Within-group changes were evaluated with paired t-tests or Wilcoxon signed-rank tests. Between-group differences in change over time were examined using repeated-measures analysis of variance (ANOVA). If assumptions of normality or sphericity were violated, non-parametric alternatives (e.g., Mann–Whitney U test for change scores) were applied.

Missing data were handled using complete-case analysis, with pairwise deletion for variable-specific missingness. The extent of missingness was summarized, and sensitivity analyses compared results with and without imputation.

Because this was a real-world retrospective study, laboratory investigations were ordered based on clinical indication rather than a standardized research protocol. Consequently, not all metabolic variables were available for all participants. Analyses were therefore conducted using variable-specific complete-case approaches, and the number of patients contributing data for each outcome is explicitly reported

### Ethical considerations

The study protocol was approved by the Research and Ethics Committee of PSMMC (HP-01-R079). All patient data were anonymized before analysis, and good clinical practice principles were followed. The requirement for informed consent was waived due to the retrospective design. The study complied with institutional guidelines and the principles of the Declaration of Helsinki.

## Results

### Baseline characteristics

A total of 664 adults with obesity were included in the analysis, of whom 299 received Liraglutide and 365 did not. Before matching, the Liraglutide group had higher median BMI compared to the non-Liraglutide group (36 [IQR, 33.00–38.00] vs. 35.00 [IQR, 32.00–37.00] kg/m²; p = 0.001), while the distribution of age, sex, and comorbidities such as hypertension, prediabetes, and hypothyroidism was similar across groups (all p > 0.050). To reduce baseline confounding, we performed 1:1 propensity score matching, yielding 280 well-matched pairs. After matching, most baseline characteristics were well balanced between groups, with most standardized mean differences (SMDs) below 0.100, indicating adequate covariate balance (Table 1).

**Table 1:** Baseline Characteristics After Propensity Score Matching (Core variables).

Variable	No Liraglutide (n = 280)	Liraglutide (n = 280)	SMD
Age (years, median (IQR))	39 (33–49)	40 (33–47)	0.06
Female sex (n (%))	207 (73.9%)	202 (72.1%)	0.04
BMI (kg/m², median (IQR))	36 (33–38)	35 (33–38)	0.05
Waist circumference (cm, median (IQR))	108 (100–115) [n = 137]	105 (98–111) [n = 114]	0.19
SBP (mmHg, median (IQR))	123 (114–130) [n = 251]	123 (112–129) [n = 253]	0.08
DBP (mmHg, median (IQR))	77 (70–81) [n = 251]	77 (70–82) [n = 253]	0.01
HbA1c (%, median (IQR))	5.7 (5.4–5.9) [n = 119]	5.6 (5.4–5.9) [n = 128]	0.17
LDL (mmol/L, median (IQR))	3.1 (2.3–3.6) [n = 98]	2.8 (2.0–3.5) [n = 113]	0.15
HDL (mmol/L, median (IQR))	1.2 (1.0–1.5) [n = 98]	1.2 (1.0–1.4) [n = 113]	0.06
Triglycerides (mmol/L, median (IQR))	1.3 (0.9–1.9) [n = 98]	1.3 (0.9–1.8) [n = 113]	−0.06
Hypertension (n (%))	23 (8.2%)	26 (9.3%)	−0.03
Prediabetes (n (%))	35 (12.5%)	37 (13.2%)	−0.02
Hypothyroidism (n (%))	43 (15.4%)	30 (10.7%)	0.13

**Note:** The numbers in the column headings (n) indicate the total number of patients in each group. Values in square brackets within cells represent the number of patients with available data for that variable, when less than the total group size.

**Abbreviations:** BMI, body-mass index; SBP, systolic blood pressure; DBP, diastolic blood pressure; HbA1c, glycated hemoglobin; LDL, low-density lipoprotein; HDL, high-density lipoprotein; and SMD, standardized mean difference. An SMD of less than 0.1 indicates adequate covariate balance between groups.

Among patients receiving Liraglutide, the median duration of treatment exposure was six months (IQR 4–9 months). During follow-up, less than 5% of patients discontinued Liraglutide. When documented, the most common reasons for discontinuation included gastrointestinal intolerance and patient preference. In the matched cohort, the median age was 40 years in both groups (IQRs, 32.00–47.00 Liraglutide and 33.00–49.00 in non-Liraglutide), with females comprising 72.14% and 73.93% of participants, respectively (SMD = 0.04). The prevalence of hypertension (9.29% vs. 8.21%), prediabetes (13.21% vs. 12.50%), and hypothyroidism (10.71% vs.15.36%) remained comparable after matching. There were no significant differences in baseline metabolic profiles, anthropometric measures, or behavioral factors. Detailed baseline comorbidities, laboratory measures, and behavioral variables are shown in the S2 and S3 Tables.

**Fig 2:**
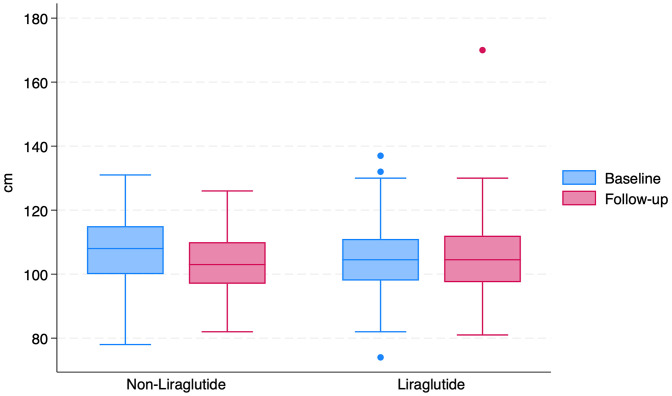
Box Plot of waist circumference at baseline and follow-up in Liraglutide and non-Liraglutide. The box plot illustrates waist circumference measurements at baseline and follow-up for each group. Within-group changes were analyzed using the Wilcoxon test, showing no significant difference in the non-Liraglutide group (P = 0.432) and the Liraglutide group (P = 0.758). Between-group comparison at follow-up using ANOVA revealed a statistically significant difference (P = 0.03).

Clinical Outcomes After Propensity Score Matching: In the matched cohort (n = 560), both groups showed statistically significant improvements in several clinical parameters following intervention. For metabolic outcomes, between-group comparisons reflect only participants with available paired measurements. Differences in sample size across outcomes are reported to maintain transparency and avoid overinterpretation of findings derived from incomplete data. Within-group analysis demonstrated reductions in BMI, systolic and diastolic blood pressure, and HbA1c in both the Liraglutide and non-Liraglutide groups (Wilcoxon p < 0.05 for all) as reported in Table 2. Between-group comparisons using repeated-measures ANOVA showed a statistically significant difference only for waist circumference (p = 0.03, Fig 2). The change in BMI did not differ significantly between groups (− 0.75 kg/m² in the Liraglutide group vs. − 0.71 kg/m² in the non-Liraglutide group; p = 0.939), although within-group reductions were statistically significant in both groups.

**Table 2:** Comparison of clinical outcomes between Liraglutide and non-Liraglutide, after propensity score matching.

Variable	Group	Baseline (n = 280)	Follow-up (n = 280)	P*-value (Wilcoxon)	P**-value (ANOVA)
BMI (kg/m², median (IQR))	Liraglutide	35 (33-38)	34.5 (31.5-38.1)	<0.001	0.939
	No Liraglutide	36 (33-38)	35.2 (31.7-38.0)	<0.001	
SBP (mmHg)	Liraglutide	123 (112–129) [n = 253]	130.2 ± 21.3	<0.001	0.182
	No Liraglutide	123 (114–130) [n = 251]	128.8 ± 20.7	0.01	
DBP (mmHg)	Liraglutide	77 (70–82) [n = 253]	70.5 ± 8.5	<0.001	0.228
	No Liraglutide	77 (70–81) [n = 251]	68.8 ± 9.2	<0.001	
HbA1c (%)	Liraglutide	5.6 (5.4–5.9) [n = 128]	5.6 ± 0.4	0.003	0.527
	No Liraglutide	5.7 (5.4–5.9) [n = 119]	5.6 ± 0.4	0.01	
FBS (mmol/L)	Liraglutide	5 (4.6–5.5) [n = 77]	4.9 ± 0.6	0.008	0.488
	No Liraglutide	5.1 (4.8–5.6) [n = 72]	5.06 ± 0.62	0.142	
Total Cholesterol (mmol/L)	Liraglutide	4.9 (4.2–5.4) [n = 114]	4.8 ± 0.9	0.491	0.353
	No Liraglutide	4.8 (4.3–5.6) [n = 95]	4.8 ± 0.9	0.225	
LDL (mmol/L)	Liraglutide	2.8 (2.0–3.5) [n = 113]	3.0 ± 0.9	0.103	0.464
	No Liraglutide	3.1 (2.3–3.6) [n = 98]	3.0 ± 0.9	0.712	
HDL (mmol/L)	Liraglutide	1.2 (1.0–1.4) [n = 113]	1.2 ± 0.3	0.473	0.053
	No Liraglutide	1.2 (1.00–1.5) [n = 98]	1.4 ± 0.3	0.052	
Triglycerides (mmol/L)	Liraglutide	1.3 (0.9–1.8) [n = 113]	1.12 (0.9-1.6)	0.181	0.567
	No Liraglutide	1.3 (0.9–1.9) [n = 98]	1.13 (0.8-1.4)	0.030	
ALT (IU/L)	Liraglutide	14 (9–22) [n = 118]	14 (10-21)	0.687	0.693
	No Liraglutide	16 (11–24) [n = 105]	16 (11-25.8)	0.319	

**Notes:** Values are expressed as mean ± SD for normally distributed data and as median (interquartile range [IQR]) for skewed data. The numbers in the column headings (n) indicate the total number of patients in each group.

**Abbreviations:** BMI, body-mass index; SBP, systolic blood pressure; DBP, diastolic blood pressure; HbA1c, glycated hemoglobin; FBS, fasting blood sugar; LDL, low-density lipoprotein; HDL, high-density lipoprotein; ALT, alanine aminotransferase; and SMD, standardized mean difference. P *values indicate the significance of changes from baseline to follow-up within each group; P** values indicate the significance of between-group differences. A P value <0.05 was considered statistically significant.

Similarly, while both groups exhibited reductions in HbA1c, fasting blood glucose, and serum triglycerides, between-group differences were not statistically significant (all p > 0.05). Lipid parameters (LDL, HDL, total cholesterol) and ALT levels remained largely unchanged or showed minor non-significant differences between groups. Additional analyses of fasting glucose, vitamin D, and behavioral or referral variables (smoking, diet, physical activity, PHQ-2 score, specialty referrals) are provided in the S2 Table.

## Discussion

In this real-world primary care cohort, repeated-measures ANOVA demonstrated a statistically significant between-group difference in waist circumference change (p = 0.03), without meaningful differences in BMI, glycemic control, blood pressure, or lipid profile. These findings highlight Liraglutide’s preferential impact on central adiposity, consistent with randomized controlled trials and meta-analyses [28–31].

Santilli et al. reported greater visceral fat reduction with Liraglutide than with lifestyle counseling alone [28], while Schmidt et al. found that combining Liraglutide with dietary education enhanced central adiposity loss compared to Liraglutide monotherapy [29]. Our findings are consistent with these observations; however, they suggest that when lifestyle care is delivered in a structured, multidisciplinary, and closely monitored manner, the additional metabolic benefits of Liraglutide may be diminished. The absence of significant between-group differences in BMI and most cardiometabolic outcomes likely reflects the high intensity of the lifestyle intervention provided to both groups, including structured dietary counseling, prescribed physical activity, and multidisciplinary follow-up. Such interventions have previously achieved metabolic benefits comparable to pharmacotherapy in high-adherence settings [32–34]. In our cohort, the lifestyle program alone yielded clinically relevant within-group improvements in anthropometric and metabolic parameters, underscoring its central role in obesity care.

Clinically, these data reinforce the value of embedding lifestyle medicine within primary care as the foundation of obesity management [35]. Pharmacotherapy may be most impactful in patients with marked central adiposity [29], poor response to lifestyle measures, or difficulty maintaining adherence to nonpharmacologic strategies [24]. Given Liraglutide’s cost and resource implications, identifying such patient subgroups is critical for optimizing care allocation. Indeed, a Taiwanese real-world study demonstrated that, despite elevated pharmacy expenses, Liraglutide use was associated with lower inpatient, ER, and overall medical costs per patient per month compared to basal insulin, supporting a targeted approach to therapy allocation [36].

These interpretations should be considered in light of the study’s methodological strengths and limitations. Strengths of this study include its real-world primary care setting, enhancing the applicability of findings to routine clinical practice, and the use of a comprehensive electronic registry with standardized measurements for anthropometric, metabolic, and behavioral variables. The large sample size and application of 1:1 propensity score matching achieved excellent baseline balance (SMD < 0.10 for most covariates), reducing confounding and strengthening internal validity. Both study groups received a structured, multidisciplinary lifestyle program, reflecting contemporary best-practice obesity care and providing a rigorous comparator for pharmacotherapy.

Limitations include its retrospective, single-center design, which may limit generalizability beyond other populations. The short median follow-up (3–6 months) precludes assessment of sustained weight loss or metabolic changes. Selection bias is possible, as only patients referred to lifestyle medicine clinics were included. The absence of detailed adherence data for dietary and physical activity prescriptions limits assessment of intervention fidelity, and no information on medication tolerability or adverse events was collected. Despite propensity matching, residual confounding from unmeasured variables cannot be excluded. Objective measures of medication adherence, such as pharmacy refill data or medication possession ratios, were not available in the electronic registry. Prescription records therefore reflect clinician intent to treat rather than confirmed adherence. This limitation is inherent to retrospective real-world studies and may have attenuated observed between-group differences. Finally, the study may have been underpowered to detect modest but clinically meaningful differences between groups, particularly in secondary outcomes.

Our findings should be interpreted as reflecting the effectiveness of Liraglutide under routine primary care conditions, rather than pharmacologic efficacy under controlled trial settings. The modest incremental benefit observed may reflect high-intensity lifestyle intervention delivered to both groups, variable medication adherence, and the relatively short follow-up period.

Future studies should assess longer-term outcomes, stratify participants by severity of obesity or comorbidity status, and incorporate patient-reported outcomes to evaluate the quality-of-life impacts. Additionally, cost-effectiveness analyses will be crucial for informing health policy in resource-limited settings.

## Conclusion

In this real-world study from a primary care lifestyle medicine clinic in Saudi Arabia, both Liraglutide plus lifestyle intervention and lifestyle intervention alone led to significant improvements in anthropometric and metabolic outcomes in adults with obesity. While Liraglutide conferred additional benefits, especially in reducing waist circumference, these did not result in clinically significant differences compared to lifestyle changes alone. These results emphasize the effectiveness of structured, multidisciplinary lifestyle care in primary care and suggest that pharmacotherapy may provide added value in selected patients. Longer-term, prospective studies are warranted to assess sustained outcomes and economic implications of combined therapy.

## Supporting information

S1 Table Variables Extracted from the Lifestyle Medicine Clinic (LMC) electronic patient registry and measurement schedule. (DOCX)

S2 Table Baseline characteristics before matching. (DOCX)

S3 Table Baseline characteristics of additional variables after matching. (DOCX)

## Abbreviations

ALT: Alanine Aminotransferase
ANOVA: Analysis of Variance
BMI: Body Mass Index
DBP: Diastolic Blood Pressure
EOSS: Edmonton Obesity Staging System
FBS: Fasting Blood Sugar
HbA1c: Glycated Hemoglobin
HDL: High-Density Lipoprotein
IPAQ: International Physical Activity Questionnaire
IQR: Interquartile Range
LMC: Lifestyle Medicine Clinic
LDL: Low-Density Lipoprotein
MASH: Metabolic Dysfunction–Associated Steatohepatitis
PHQ-2/ PHQ-9: Patient Health Questionnaire-2/ −9
PSMMC: Prince Sultan Military Medical City
SBP: Systolic Blood Pressure
SD: Standard Deviation
SMD: Standardized Mean Difference
TG: Triglycerides
25(OH)D: 25-Hydroxyvitamin D
