# Supplementary material for: Clinical and metabolic profile of adults with obesity attending lifestyle medicine clinics
Source: PLoS One. 2026 Feb 2;21(2):e0342153. doi: 10.1371/journal.pone.0342153 (PMC12863516; doi:10.1371/journal.pone.0342153)
Supplement: S1 Table — (DOCX) [file pone.0342153.s001.docx]

**S1 Table.** **Variables Extracted from the Lifestyle Medicine Clinic (LMC) Electronic Patient Registry and Measurement Schedule**

| **Variable** | **Definition / Measurement** | **Unit / Category** | **Baseline** | **3 months** | **6 months** |
| --- | --- | --- | --- | --- | --- |
| Age | Age at baseline | Years (14–75) | ✓ |  |  |
| Sex | Biological sex | Male / Female | ✓ |  |  |
| Blood Pressure | Systolic and diastolic BP, seated after rest | mmHg | ✓ | ✓ | ✓ |
| Weight | Measured without shoes, light clothing | kg | ✓ | ✓ | ✓ |
| Height | Measured without shoes using stadiometer | m | ✓ |  |  |
| Body Mass Index (BMI) | Weight (kg) ÷ height (m²) | kg/m² | ✓ | ✓ | ✓ |
| Waist Circumference (WC) | Midpoint between lower rib and iliac crest; Men >102 cm, Women >88 cm = abdominal obesity | cm | ✓ | ✓ | ✓ |
| Edmonton Obesity Staging System (EOSS) | Clinical staging (0–4) | Stage 0–4 | ✓ |  |  |
| Change in WC | WC follow-up – WC baseline | cm |  | ✓ | ✓ |
| Change in Weight | Weight follow-up – Weight baseline | kg |  | ✓ | ✓ |
| Total Body Weight Loss (%) | [(Weight follow-up – baseline weight) ÷ baseline weight] × 100 | % |  | ✓ | ✓ |
| Smoking Status | Self-reported | Current / Former / Never | ✓ |  |  |
| Chronic Medical Conditions | Hypertension, hyperlipidemia, hypothyroidism, MASH, bronchial asthma | Yes / No | ✓ |  |  |
| PHQ-2 Score | Patient Health Questionnaire-2 | Score 0–6 | ✓ | ✓ | ✓ |
| HbA1c | Glycated hemoglobin | % | ✓ | ✓ | ✓ |
| LDL-C | Low-density lipoprotein cholesterol | mmol/L | ✓ | ✓ | ✓ |
| HDL-C | High-density lipoprotein cholesterol | mmol/L | ✓ | ✓ | ✓ |
| Triglycerides (TG) | Serum triglycerides | mmol/L | ✓ | ✓ | ✓ |
| ALT | Alanine aminotransferase | U/L | ✓ | ✓ | ✓ |
| Vitamin D (25(OH)D) | Serum 25-hydroxyvitamin D | nmol/L or ng/mL | ✓ | ✓ | ✓ |

Abbreviations: BP = blood pressure; BMI = body mass index; WC = waist circumference; EOSS = Edmonton Obesity Staging System; MASH = metabolic dysfunction–associated steatohepatitis; PHQ-2 = Patient Health Questionnaire-2; HbA1c = glycated hemoglobin; LDL-C = low-density lipoprotein cholesterol; HDL-C = high-density lipoprotein cholesterol; TG = triglycerides; ALT = alanine aminotransferase; 25(OH)D = 25-hydroxyvitamin D.
