# Supplementary material for: Clinical and metabolic profile of adults with obesity attending lifestyle medicine clinics
Source: PLoS One. 2026 Feb 2;21(2):e0342153. doi: 10.1371/journal.pone.0342153 (PMC12863516; doi:10.1371/journal.pone.0342153)
Supplement: S2 Table — (DOCX) [file pone.0342153.s002.docx]

**S2 Table.** **Baseline Characteristics Before Matching**

| **Variable** | **No Liraglutide (n=365)** | **Liraglutide (n=299)** | **P-value** |
| --- | --- | --- | --- |
| Age (years, median (IQR)) | 40 (33–49) | 40 (32–47) | 0.161 |
| Female sex (n (%)) | 271 (74.3%) | 218 (72.9%) | 0.697 |
| BMI (kg/m², median (IQR)) | 35 (32–37) | 36 (33–38) | 0.001 |
| Waist circumference (cm, median (IQR)) | 106 ± 11 | 105 ± 10 | 0.597 |
| SBP (mmHg, median (IQR)) | 123 (114–130) | 122 (112–129) | 0.342 |
| DBP (mmHg, median (IQR)) | 77 (70–82) | 76 (70–81) | 0.412 |
| HbA1c (%, median (IQR)) | 5.7 ± 0.4 | 5.6 ± 0.4 | 0.083 |
| LDL (mmol/L, median (IQR)) | 3.1 (2.3–3.6) | 2.77 (2.0–3.5) | 0.138 |
| HDL (mmol/L, median (IQR)) | 1.2 (1.0–1.5) | 1.2 (1.0–1.4) | 0.353 |
| Triglycerides (mmol/L, median (IQR)) | 1.3 (0.9–2.0) | 1.3 (0.94–1.78) | 0.724 |
| Hypertension (n (%)) | 36 (9.9%) | 26 (8.7%) | 0.607 |
| Prediabetes (n (%)) | 51 (14.0%) | 38 (12.7%) | 0.634 |
| Hypothyroidism (n (%)) | 54 (14.8%) | 30 (10.0%) | 0.066 |
| Fatty liver (n (%)) | 1/353 (0.3%) | 3/296 (1.0%) | 0.336 |
| Edmonton obesity scoring system (EOSS) stage  Stage 0  Stage 1  Stage 2  Stage 3  Stage 4 | 224 (61.4%) 95 (26.0%) 44 (12.0%) 1 (0.3%) 1 (0.3%) | 197(65.9%) 62 (20.7%) 40 (13.4%) 0 0 | 0.315 |
| FBS (median (IQR)) | 5.2 (4.8- 5.6) | 5.1 (4.6- 5.5) | 0.084 |
| Total cholesterol (median (IQR)) | 4.9 (4.4-5.6) | 4.9 (4.1-5.4) | 0.109 |
| ALT (median (IQR)) | 16 (10- 24) | 14 (9-22) | 0.200 |

The numbers in the column headings (n) represent the total number of patients in each group. Abbreviations: BMI: Body Mass Index, SBP: Systolic Blood Pressure, DBP: Diastolic Blood Pressure, HbA1c: Hemoglobin A1c, FBS: Fasting Blood Sugar, LDL: Low-Density Lipoprotein, HDL: High-Density Lipoprotein, ALT: Alanine Aminotransferase. P-value: Represents the statistical significance of the difference between groups. A p-value < 0.05 indicates significance.
