# Supplementary material for: Clinical and metabolic profile of adults with obesity attending lifestyle medicine clinics
Source: PLoS One. 2026 Feb 2;21(2):e0342153. doi: 10.1371/journal.pone.0342153 (PMC12863516; doi:10.1371/journal.pone.0342153)
Supplement: S3 Table — (DOCX) [file pone.0342153.s003.docx]

**S3 Table. Baseline Characteristics of Additional Variables After Matching**

| **Variable** | **No Liraglutide (n=280)** | **Liraglutide (n=280)** | **SMD** |
| --- | --- | --- | --- |
| Smoking, n (%) | 14(5.5%) [n=254] | 8(3.1%) [n=259] | 0.12 |
| Diet, n (%) | 64 (22.9%) | 81 (28.9%) | -0.14 |
| Exercise, n (%) | 59 (21.1%) | 82 (29.3%) | -0.19 |
| Lifestyle modification, n (%) | 272 (97.1%) | 275 (98.2%) | -0.07 |
| Dietitian referral, n (%) | 234 (83.6%) | 227 (81.1%) | 0.07 |
| Psychiatry referral, n (%) | 0 (0.0%) | 2 (0.7%) | -0.12 |
| Psychology referral, n (%) | 1 (0.4%) | 1 (0.4%) | 0.00 |
| Endocrine referral, n (%) | 1 (0.4%) | 1 (0.4%) | 0.00 |
| Clinical pharmacy referral, n (%) | 6 (2.1%) | 9 (3.2%) | -0.07 |
| Sleep clinic referral, n (%) | 3 (1.1%) | 2 (0.7%) | 0.04 |
| Depression (PHQ2 ≥3), n (%) | 15 (5.4%) | 14 (5.0%) | 0.02 |
| ALT (U/L), median (IQR) | 16 (11–24) [n=105] | 14 (9–22)[n=118] | 0.16 |
| Vitamin D (nmol/L), median (IQR) | 43 (30–57) [n=113] | 39 (28–53) [n=121] | 0.14 |
| Fatty liver, n (%) | 1 (0.4%) [n=273] | 2 (0.7%) [n=278] | -0.04 |
| Edmonton obesity scoring system (EOSS) stage  Stage 0  Stage 1  Stage 2  Stage 3  Stage 4 | 174 (62.1 %) 75 (26.8%) 31 (11.1%) 0 0 | 182 (65.0%) 58 (20.7%) 40 (14.3%) 0 0 | -0.01 |
| FBS, median (IQR) | 5.1 (4.8- 5.6) [n= 72] | 5 (4.6- 5.5) [n=77] | 0.16 |
| Total cholesterol, median (IQR) | 4.8 (4.3- 5.6) [n=95] | 4.9 (4.2-5.4) [n=114] | 0.19 |

The numbers in the column headings (n) represent the total number of patients in each group. The numbers in square brackets within cells [n] indicate the actual number of patients with available data for that specific variable, when less than the total group size (n). Abbreviations: ALT: Alanine Aminotransferase, FBS: Fasting Blood Sugar. SMD: Standardized Mean Difference. An SMD < 0.1 indicates adequate covariate balance, suggesting that the groups are comparable on that variable.
